# Characterization of the obstructive site and type of collapse in obstructive sleep apnea and oropharyngeal dysphagia: Letter to the editor

**DOI:** 10.1590/2317-1782/e20250379en

**Published:** 2026-07-03

**Authors:** Isabela Cristina Monteiro de Lira, Hilton Justino da Silva, Danielle Andrade da Cunha, Leandro de Araújo Pernambuco, Luciana Moraes Studart-Pereira

**Affiliations:** 1 Departamento de Fonoaudiologia, Universidade Federal de Pernambuco – UFPE - Recife (PE), Brasil.

Dear editor,

This letter discusses the article entitled “Obstructive Sleep Apnea: characterization of the obstructive site and type of collapse”^(1)^. The study aimed to describe and illustrate the locations and types of upper airway (UA) collapse during sleep to contribute to the understanding of the pathophysiology of obstructive sleep apnea (OSA), eligibility for individualized therapeutic procedures, and guidance for orofacial myofunctional therapy. The authors selected 11 cases of patients from the otolaryngology outpatient clinic of the Samaritano Hospital in São Paulo, Brazil, with a diagnosis of OSA and who underwent drug-induced sleep endoscopy (DISE).

The results showed a greater number of cases with velopharyngeal collapse, followed by oropharyngeal, lingual, and, lastly, epiglottic collapses. Despite the small sample size, we congratulate the authors on their excellent and relevant work, which includes DISE images and offers a valuable contribution to the understanding of the pathophysiology of OSA and the structures involved in collapse sites^(1)^.

In describing obstructive sites in OSA, although it does not explicitly discuss dysphagia, the article opens relevant possibilities for reflection in this area as well. Collapses occur in structures and regions important for swallowing function, such as the base of the tongue, the velopharyngeal sphincter, and the epiglottis, which, during the hyolaryngeal forward and upward excursion, folds over the glottic space and contributes to the closure of the laryngeal vestibule, coinciding with the relaxation of the cricopharyngeal muscle and the opening of the upper esophageal sphincter (UES) for the passage of the food bolus^(2,3)^.

In its pharyngeal phase, swallowing is a pressure-driven event that requires complex coordination of muscle contraction to ensure safe swallowing. During bolus transport, different pressure events are generated at the base of the tongue, velopharynx, and UES, which vary according to the volume of the bolus, in order to safely propel it in the correct direction. These regions coincide with the sites described in the study^(2)^.

The relationship between OSA and orofacial function impairments is already known, reinforcing the interdependence between sleep quality, respiratory function, and orofacial myofunctional performance^(4,5)^. Oropharyngeal dysphagia stands out among orofacial function impairments, since about 15% of patients diagnosed with OSA present symptoms of dysphagia^(6)^. People with OSA may have ineffective oral control, premature posterior escape, a deficit in bolus propulsion, delayed onset of the pharyngeal phase, multiple swallows, pharyngeal residues, and laryngeal penetration^(6,7)^. However, in clinical settings, dysphagia is often an underestimated and poorly recognized sequela associated with OSA^(8)^.

The association between OSA and oropharyngeal dysphagia is still marked by uncertainties, keeping the topic under discussion. It is already known that vibratory trauma caused by obstruction and snoring can be one of these factors, as the presence of edema and lesions caused by repeated events can alter the sensitivity of sensory receptors present in the pharynx. These receptors are essential for preparing the bolus, the proper positioning of oropharyngeal structures, and the modulation of muscle contraction^(9)^. Therefore, the cited article has an important impact on the clinical reasoning and therapeutic planning of professionals who work with this population.

In this context, it is relevant to consider that patients with OSA often do not recognize or report swallowing impairment. OSA is a highly prevalent condition, affecting approximately 936 million adults worldwide^(10)^, which makes the possibility of underreporting dysphagia symptoms even more worrying. This may result from the difficulty in perceiving subtle signs of dysphagia or even the tendency to consider them irrelevant. The discrepancy between the patient's subjective perception and the presence of the swallowing disorder shows that dysphagia may remain masked in this population^(11)^.

The findings regarding the sites and types of UA collapse, combined with the pathophysiological reasoning proposed here, point to a possible interface with dysphagia. Future investigations could examine this relationship objectively by associating, for instance, the assessment of swallowing with DISE. Hence, it is essential to carry out systematic swallowing assessments to identify impairments early and implement appropriate therapeutic interventions. This condition can lead to serious complications such as malnutrition, dehydration, and aspiration pneumonia, directly impacting quality of life and increasing the demand on already scarce health resources^(12,13)^. Similarly, attention should be paid to investigating the sleep of patients with swallowing complaints, given the existing interface between these disorders.
