# Supplementary material for: Characterization of the obstructive site and type of collapse in obstructive sleep apnea and oropharyngeal dysphagia: Letter to the editor
Source: Codas. 2026 Jul 3;38(4):e20250379. doi: 10.1590/2317-1782/e20250379en (PMC13330936; doi:10.1590/2317-1782/e20250379en)
Supplement: Translation

# Translation: Caracterização do sítio obstrutivo e tipo de colapso na Apneia Obstrutiva do Sono e a Disfagia Orofaríngea: Carta ao Editor

**DOI:** 10.1590/2317-1782/e20250379pt

**Authors:** Isabela Cristina Monteiro de Lira, Hilton Justino da Silva, Danielle Andrade da Cunha, Leandro de Araújo Pernambuco, Luciana Moraes Studart-Pereira

**Affiliations:** 1 Departamento de Fonoaudiologia, Universidade Federal de Pernambuco – UFPE - Recife (PE), Brasil.

Caro Editor,

O artigo intitulado “Apneia Obstrutiva do Sono: caracterização do sítio obstrutivo e tipo de colapso” é o tema de discussão nesta carta^(1)^. Nesse estudo, o objetivo foi descrever e ilustrar os locais e tipos de colapso da via aérea superior (VAS) durante o sono, a fim de contribuir para a compreensão da fisiopatologia da Apneia Obstrutiva do Sono (AOS), elegibilidade de procedimentos terapêuticos individualizados e direcionamento para terapêutica miofuncional orofacial. Foram selecionados 11 casos de pacientes do ambulatório de Otorrinolaringologia do Hospital Samaritano de São Paulo com diagnóstico de AOS e que realizaram a endoscopia do sono induzida por drogas (DISE).

Os resultados demonstraram maior número de casos com colapso no sítio velofaríngeo, seguido pelos colapsos orofaríngeo, lingual e, por fim, epiglótico. Apesar da pequena amostra, congratulamos os autores pelo excelente e relevante trabalho, que conta com imagens da DISE, e oferece uma valiosa contribuição para a compreensão da fisiopatologia da AOS e das estruturas envolvidas nos sítios de colapsos^(1)^.

Ao descrever os sítios obstrutivos na AOS, embora não discuta explicitamente a disfagia, o artigo abre possibilidades relevantes de reflexão também para essa área, visto que os colapsos ocorrem em estruturas e regiões importantes para a função da deglutição, como a base de língua, o esfíncter velofaríngeo e a epiglote, que, durante a excursão hiolaríngea, para frente e para cima, dobra-se sobre o espaço glótico e contribui para o fechamento do vestíbulo laríngeo, coincidindo com o relaxamento do músculo cricofaríngeo e a abertura do esfíncter esofágico superior (EES) para passagem do bolo alimentar^(2,3)^.

Assim, em sua fase faríngea, a deglutição é um evento impulsionado pela pressão, que requer coordenação complexa da contração muscular para garantir uma deglutição segura. Durante o transporte do bolo alimentar, são gerados diferentes eventos de pressão na base de língua, velofaringe e EES, que variam conforme o volume do bolo, a fim de impulsioná-lo com segurança para a direção correta. Essas regiões coincidem com os sítios descritos no estudo^(2)^.

Já é sabida a relação entre AOS e alterações nas funções orofaciais, reforçando a interdependência entre a qualidade do sono, a função respiratória e o desempenho miofuncional orofacial^(4,5)^. Dentre as alterações nas funções orofaciais, pode-se destacar a disfagia orofaríngea, uma vez que, cerca de 15% dos pacientes diagnosticados com AOS apresentam sintomas de disfagia^(6)^. Pessoas com AOS podem apresentar controle oral ineficaz, escape posterior prematuro, déficit na propulsão do bolo alimentar, atraso no início da fase faríngea, deglutições múltiplas, resíduos faríngeos e penetração laríngea^(6,7)^. No cenário clínico, entretanto, a disfagia costuma ser uma sequela subestimada e pouco reconhecida em associação à AOS^(8)^.

A associação entre AOS e disfagia orofaríngea ainda é marcada por incertezas, mantendo o tema em discussão. O que já se conhece é que o trauma vibratório causado pela obstrução e pelo ronco pode ser um desses fatores, pois a presença do edema e das lesões provocadas pelos eventos repetidos, podem alterar a sensibilidade dos receptores sensoriais presentes na faringe. Esses receptores são essenciais para o preparo do bolo alimentar, o posicionamento adequado das estruturas orofaríngeas e a modulação da contração muscular^(9)^. Por isso, o artigo citado tem importante impacto no raciocínio clínico e planejamento terapêutico dos profissionais que irão atuar com essa população.

Nesse contexto, é relevante considerar que pacientes com AOS frequentemente não reconhecem ou não relatam alterações relacionadas à deglutição. A AOS é uma condição de alta prevalência, que afeta cerca de 936 milhões de adultos em todo o mundo^(10)^. O que torna mais preocupante a possibilidade de subnotificação dos sintomas de disfagia, que pode decorrer da dificuldade em perceber sinais sutis da disfagia ou mesmo da tendência a considerá-los como irrelevantes. A discrepância entre a percepção subjetiva do paciente e a presença do distúrbio de deglutição, evidencia que a disfagia pode permanecer mascarada nessa população^(11)^.

Os achados referentes aos locais e tipos de colapso da via aérea superior, aliados ao raciocínio fisiopatológico aqui proposto, apontam para uma possível interface com a disfagia. Investigações futuras poderiam examinar essa relação de forma objetiva, por exemplo, associando a avaliação da deglutição à DISE. Nessa perspectiva, torna-se fundamental a realização de avaliações sistemáticas da deglutição, a fim de identificar precocemente alterações e possibilitar a implementação de intervenções terapêuticas adequadas, uma vez que essa condição pode acarretar complicações graves, como desnutrição, desidratação e pneumonia aspirativa, o que impacta diretamente a qualidade de vida e aumenta a demanda sobre os já escassos recursos de saúde^(12,13)^. Da mesma forma, deve-se atentar para investigar o sono de pacientes com queixas de deglutição, diante da interface existente entre esses distúrbios.
